# High-precision mass and $\beta ^-$ decay *Q*-value measurement of $^{129}$Sb and its isomer

**DOI:** 10.1140/epja/s10050-026-01883-8

**Published:** 2026-06-23

**Authors:** M. Winter, Z. Ge, T. Eronen, A. Kankainen, P. Ascher, O. Beliuskina, A. de Roubin, M. Flayol, M. Gerbaux, W. Gins, S. Grévy, M. Hukkanen, A. Husson, A. Jaries, A. Jokinen, I. D. Moore, D. A. Nesterenko, A. Raggio, J. Ruotsalainen, J. Romero, M. Stryjczyk, V. A. Virtanen, A. Zadvornaya

**Affiliations:** 1 Department of Physics, University of Jyväskylä, P.O. Box 35, 40014 Jyväskylä, Finland; 2 Université de Bordeaux, CNRS/IN2P3, LP2I Bordeaux, UMR 5797, 33170 Gradignan, France; 3 Department of Physics, University of Liverpool, Liverpool, L69 7ZE UK; 4 Present Address: Université de Caen Normandie, CNRS/IN2P3, LPC Caen UMR6534, 14000 Caen, France; 5 Present Address: Max-Planck-Institut Für Kernphysik, 69117 Heidelberg, Germany; 6 Present Address: KU Leuven, Instituut voor Kern- en Stralingsfysica, 3001 Leuven, Belgium; 7 Present Address: GSI Helmholtzzentrum für Schwerionenforschung GmbH, 64291 Darmstadt, Germany; 8 Present Address: Institut Laue-Langevin, 38042 Grenoble, France

## Abstract

The $\beta ^-$ decay *Q* value and mass excess of $^{129}$Sb were measured using the JYFLTRAP double Penning trap. The $^{129}$Sb isotopes were produced and separated on-line using the IGISOL facility to precisely measure these quantities. The ground-state-to-ground-state $Q_\beta$ value was determined to be 2411.3(12) keV, which is a factor of $\sim$ 18 improvement in precision and 36(21) keV ($\approx$ 1.7$\sigma$) higher than the adopted value in the most recent Atomic Mass Evaluation AME2020. With this new $Q_\beta$, the five branches previously considered as potential ultra-low-$Q^*$ $\beta ^-$ from the ground state to excited levels in $^{129}$Te, ranging from 2360 to 2380 keV, are refined to have $Q^*_\beta > 31$ keV. This places them well outside the ultra-low regime required for enhanced sensitivity in future electron antineutrino mass experiments, definitively ruling them out as viable candidates. With the new ground-state mass, we also revised the mass-excess value for the isomeric state $^{129m}$Sb ($19/2^-$), which is a well-known candidate for Nuclear Excitation by Electron Capture (NEEC). The new value reveals a potential ultra-low $\beta$ transition from $^{129m}$Sb with $Q^*_\beta =3.3(13)$ keV. However, the spin-parity is most likely unfavorable for the beta transition. Furthermore, the newly determined mass excess of $^{129}$Sb, $-\,84{,}593.6(14)$ keV, is used to deduce the $Q_{\beta }$ values of ground and isomeric states of $^{129}$Sn, which are relevant for the *r*-process nucleosynthesis models and kilonova.

## Introduction

Nuclear isomers are metastable excited states of atomic nuclei and play a pivotal role in nuclear physics, astrophysics and applications [1–3]. There are more than 1900 known isomers with a half-life longer than 100 ns [4, 5]. They can significantly influence nucleosynthesis processes, particularly the rapid neutron-capture process (*r*-process) [6] in extreme environments, such as neutron star mergers, see e.g. [7].

The isomers with astrophysical significance are sometimes referred to as astromers in the astrophysical contexts [8]. Astromers can alter decay rates, impacting the production of heavy elements and affecting isotopic distributions. Moreover, the radioactive decay energies are typically higher than for the ground states, affecting the electromagnetic signatures of kilonovae [9]. Understanding the isomer properties, including precise energy levels, half-lives, spin-parity assignments, and decay pathways, is crucial for refining nucleosynthesis models and interpreting multi-messenger astronomical observations. Isobars at $A=129$ have been pointed out as relevant for the *r* process, in terms of their isomers [8] and the use of $^{129}$I radioisotope abundance measured in meteorites to constrain the *r*-process site [10].

Isomers are heavily involved in the decay chain $^{129}$Sn–$^{129}$Sb–$^{129}$Te-$^{129}$I and can potentially affect the electromagnetic signal detected from kilonovae [8, 9]. $^{129}$Sn, $^{129}$Sb and $^{129}$Te all have at least two long-lived states, which differ in half-life (see Table 1) and decay patterns [11]. In addition, $^{129}$Sn and $^{129}$Sb have short-lived $\upmu$s isomers [4]. For $^{129}$Te, the population of the 33.6(1)-d isomer can potentially affect the kilonova signal delays as the decay energy is released at a much slower rate as compared to the 69.6(3)-min ground state [8]. The higher-spin isomeric states are selectively populated in the decay chains at $A=129$. For example, the $(19/2^-)$ isomer in $^{129}$Sb is fed only by the $^{129}$Sn $11/2^-$ isomer $\beta$ decay and it, in turn, populates the $11/2^-$ isomer in $^{129}$Te via its $\beta$ decay [11]. While the excitation energy of the $(19/2^-)$ isomer is very well known, $E_x=1851.31(6)$ keV, its mass-excess and therefore the $Q_{\beta }$ values are known with a modest precision of 21 keV [4]. Moreover, the high-spin isomeric states in $^{129}$Sn, $^{129}$Sb and $^{129}$Te are strongly populated in fission [12] that can also affect the population pattern in the *r* process.

**Fig. 1:**
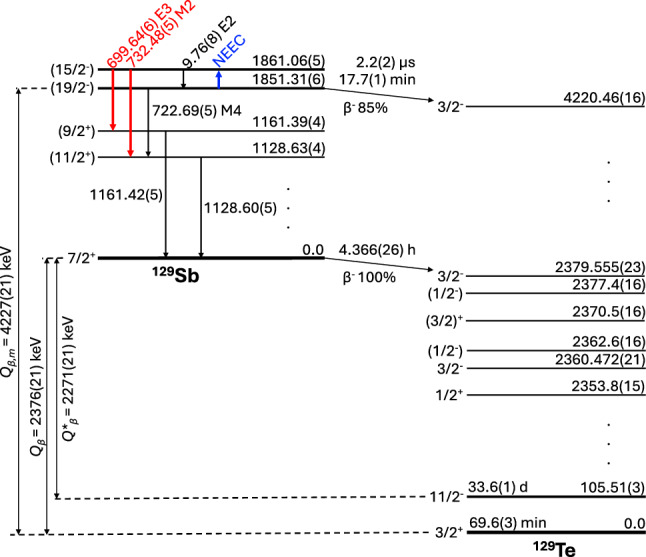
Partial level scheme for $^{129}$Sb and its daughter $^{129}$Te. The isomeric $(19/2^-)$ state at 1851 keV in $^{129}$Sb can be excited to a short-lived $(15/2^-)$ state via electron capture. This state then decays mainly via *M*2 transition to the $(11/2^+)$ state at 1129 keV, giving a clear signature of the NEEC process. The high-lying excited levels of $^{129}$Te are potential excited states for ultra-low $Q^*$-value $\beta$ decays based on the literature values [11]

The $(19/2^-)$ isomer in $^{129}$Sb has also been proposed to be a suitable candidate for the Nuclear Excitation by Electron Capture (NEEC), which is a time-reversed process for the internal conversion [13, 14]. It can take place in highly-charged ions when an electron is captured and excites the nucleus. While the first experimental evidence for NEEC [15] was later disputed [16, 17], NEEC has emerged as a powerful tool for manipulating and characterizing isomers, offering potential applications in nuclear-optical frequency standards and next-generation clocks, but requiring contamination-free experimental conditions to ensure accuracy, see e.g. [3, 18]. In $^{129}$Sb, NEEC would lead to the $(15/2^-)$, 2.2(2) $\upmu$s isomeric state at 1861.06(5) keV, around 10 keV above the $(19/2^-)$ isomer [19, 20], as indicated in Fig. 1. The 2.2(2)-$\upmu$s isomer further de-excites mainly via *M*2 and *E*3 transitions to the lower-lying states. *M*2 gamma-ray transition would give a clear signature of the NEEC process. In astrophysical conditions, this route to deplete the isomer could also affect the isomer to ground-state ratio of $^{129}$Sb.

Furthermore, $$^{129}$$Sb has been identified as a potential ultra-low $$Q_{\beta }^*$$-value candidate for ground state decay to several selected excited states in the daughter nucleus $$^{129}$$Te. Such ultra-low $$Q_{\beta }^*$$ decays are actively sought after as they serve as interesting candidates for neutrino physics studies [21–32]. There are several excited states in $$^{129}$$Te with energies close to the ground-to-ground state $$Q_{\beta }(^{129}$$Sb$$)=2376(21)$$ keV, as well as a potential one with excitation energy close to the isomer-to-ground state $$Q^*_{\beta }(^{129m}$$Sb$$)=4227(21)$$ keV [33]. These excited states are illustrated in Fig. 1.

To evaluate whether any of these could serve as a final state for an ultra-low beta decay, the $$Q_{\beta }$$ value has to be measured much more precisely. Currently, the mass of $$^{129}$$Sb in the Atomic Mass Evaluation 2020 (AME20) [33] is based on the *Q* values of the $${\beta ^-}$$-decay of $$^{129}$$Sb [34] and the $$^{130}$$Te$$(d,^3$$He$$)^{129}$$Sb reaction [35]. In this work, we present the first direct mass measurement of the $$^{129}$$Sb ground state and its beta-decay *Q* value.

## Experimental method

The experiment was performed at the Ion Guide Isotope Separator On-Line (IGISOL) facility [36] at the University of Jyväskylä, Finland, in April 2021. The radioactive ion beam was produced with proton-induced fission on a $^{nat}\text {U}$ target, using a 30-MeV proton beam. The produced ions were stopped in a helium-filled gas cell before being guided out by the gas flow into a sextupole ion guide [37]. The ions were accelerated to 30 *q*kV and separated by their mass-to-charge ratio *m*/*q* with a 55-degree dipole magnet. The ion beam was then cooled and bunched with the radiofrequency quadrupole cooler-buncher RFQ-CB [38, 39] and sent to the JYFLTRAP double Penning trap mass spectrometer [40].

**Fig. 2:**
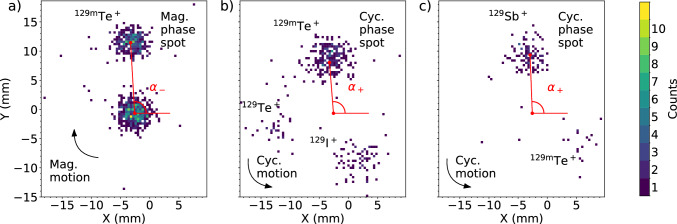
**a** The reference ion, $^{129m}$Te$^{+}$, center and magnetron phase ion spots on the two-dimensional position-sensitive MCP detector (2D-MCP) using the PI-ICR technique, with the accumulation time $t_\text {acc} = 415~\text {ms}$. The polar angle between the reference phase, the center of the trap and magnetron phase spot is marked with $\alpha _-$. **b** The cyclotron motion phase projection of $^{129m}$Te$^{+}$ ions on the 2D-MCP, with the respective polar angle $\alpha _+$. The cyclotron frequency $\nu _c$ is deduced from the angle difference between the magnetron and the cyclotron phase spots relative to the center spot. **c** The cyclotron motion phase projection for $^{129}$Sb$^{+}$ ions on the 2D-MCP, with $t_\text {acc} = 415~\text {ms}$. We note that the $^{129}$Sb$^m$ is well separated from the ground state, both having roughly similar fission yields [12]

A mass-selective buffer-gas cooling technique [41] was used to purify and center the ions in the first (preparation) trap of JYFLTRAP. First, magnetron excitation was applied to excite all ions to a larger radius, after which the ions of interest were centered when the applied quadrupolar excitation frequency matched the ion’s free-space cyclotron frequency $\nu _c = \nu _- + \nu _+= qB/(2\pi m)$, where $\nu _-$ and $\nu _+$ are the magnetron and reduced cyclotron frequencies and *B* is the magnetic field strength. The quadrupolar pulse was applied for 120 ms. After this the ions of interest were transferred to the second (measurement) trap of JYFLTRAP. To enhance the purity of the transferred ions, after around 8 ms in the second trap, the ions were transferred back to the first trap, where the quadrupolar excitation was once again applied, this time for 150 ms. After this, the ions of interest were injected to the second trap again, where the phase-imaging ion-cyclotron-resonance (PI-ICR) technique [42–45] was used to measure the free-space cyclotron frequency.

In the PI-ICR technique, the ion’s cyclotron frequency is determined from its radial eigenfrequencies $\nu _-$ and $\nu _+$ using two timing patterns in which the ions’ radial eigenmotions were excited using radio-frequency (RF) electric fields. In both patterns, a dipolar pulse with frequency $\nu _-$ was used to reduce the initial magnetron motion of the ions to center the ions, followed by a dipolar RF pulse with frequency $\nu _+$ to excite the reduced cyclotron motion. For the magnetron phase determination, the induced cyclotron motion was then converted immediately to magnetron motion by a quadrupolar pulse with frequency $\nu _c$. After an accumulation time $t_{acc}$, the ions were projected onto the 2D-MCP, where the positions of the ions were determined. For the phase of the cyclotron motion, the order of the converting quadrupolar pulse and the accumulation time were reversed. The center spot was obtained by extracting the centered ions without other excitations.

The cyclotron frequency was then calculated from the difference between the polar angles of the reduced cyclotron $(\alpha _+)$ and magnetron $(\alpha _-)$ spots with respect to the center spot:

1 $$\begin{aligned} \nu _c = \nu _+ + \nu _-= \frac{(\alpha _+ - \alpha _-) + 2\pi n_c}{2\pi t_{acc}}, \end{aligned}$$

where $n_{c}$ is the number of complete periods of the free cyclotron motion during the accumulation time $t_{acc}$ at frequency $\nu _c$.

The ratio *r* of the mass of the ion of interest $m_\text {ioi}$ to the mass of the reference ion $m_\text {ref}$ was obtained by measuring the cyclotron frequencies of the ion of interest $\nu _{c,\text {ioi}}$ and the reference ion $\nu _{c,\text {ref}}$:

2 $$\begin{aligned} r = \frac{m_\text {ioi}}{m_\text {ref}} = \frac{\nu _{c,\text {ref}}}{\nu _{c,\text {ioi}}}. \end{aligned}$$

**Fig. 3:**
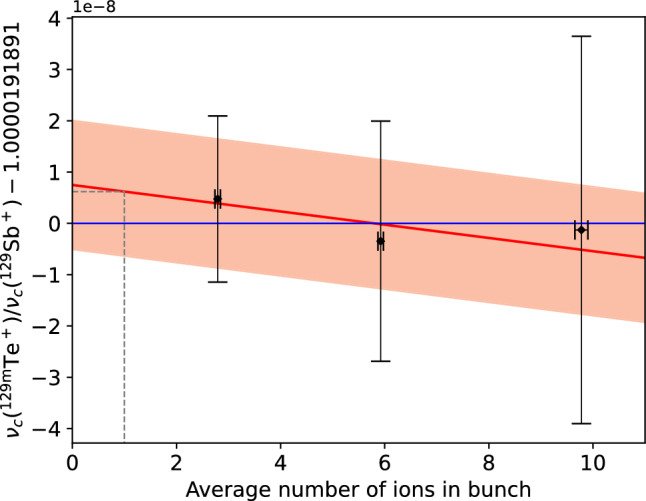
Cyclotron frequency ratio of $^{129\text {m}}\text {Te}^+$ and $^{129}\text {Sb}^+$ as a function of the number of ions in the second trap for the second measurement (see Fig. 4). The detected number of ions per bunch was corrected according to the efficiency of the detector. The red line with the shaded area represents a linear fit with $1\sigma$ uncertainty. The blue line is the mean frequency value of the three values

To determine the mass of $^{129}$Sb, two frequency ratios between $^{129}\text {Sb}^+$ and the reference $^{129m}\text {Te}^+$ ($E_x = 105.51~\text {keV}$) were measured over two hours using the phase accumulation time of 415 ms. The accumulation time was selected to ensure that the spots of the ion of interest and the reference ion were well separated from other contaminant spots (Fig. 2). The effect of magnetic field fluctuation over time was reduced by interleavedly measuring the ion of interest and the reference ion, in total 54 rounds for the ion of interest and 52 rounds for the reference ion. The frequency $\nu _c$ of the the reference was linearly interpolated to the ion of interest’s measurement time. The magnetic field fluctuation of JYFLTRAP over time has been measured to be $\delta B/(B\delta t) = 2.01 \times 10^{-12}~\textrm{min}^{-1}$ [45]. Since each interleaved cycle lasted only a few minutes, the magnetic-field drift contributes negligibly compared to other sources of uncertainty. A count-rate class analysis [45–47] did not indicate any significant dependence due to ion–ion interactions, as shown in Fig. 3.

**Table 1:** The mass-excess ($ME_{\text {JYFL}}$) and ($Q_{\beta , \text {JYFL}}$) values for $^{129}$Sb, determined from the measured frequency ratio $\overline{r}=\nu _{c}(^{129m}Te)/\nu _{c}(^{129}Sb)$ between the ground state of $^{129}$Sb and the isomeric state $^{129m}$Te, are shown in comparison with the AME2020 [33] values. The table also lists half-lives, spin-parities ($J^{\pi }$), mass excesses and excitation energies ($E_x$) of the relevant long-lived ($T_{1/2}\ge 1$ ms) states in $^{129}$Sn, $^{129}$Sb and $^{129}$Te according to [4]. Revised $Q_{\beta }$ values are also obtained for $^{129}$Sn and $^{129m}$Sn by using the mass-excess value of $^{129}$Sb determined in this work and the values from Ref.  [48] for the $^{129}$Sn states, marked with $^a$

Nuclide	$T_{1/2}$	$J^{\pi }$	$E_x$ (keV)	$ME_\text {AME20}$ (keV)	$\overline{r}$	$ME_\text {JYFL}$ (keV)	$Q_{\beta ,\text {JYFL}}$ (keV)	$Q_{\beta ,\text {AME20}}$ (keV)
$^{129}$Te	69.6(3) min	$3/2^+$	0	${}-87004.9(7)$				
$^{129m}$Te	33.6(1) d	$11/2^-$	105.51(3)	${}-86899.4(7)$				
$^{129}$Sb	4.366(26) h	$7/2^+$	0	${}-84629(21)$	1.000019203(10)	${}-84593.6(14)$	2411.3(12)	2376(21)
$^{129m}$Sb	17.7(1) min	$(19/2^-)$	1851.31(6)	${}-82778(21)$		$-82742.3(14)$	4262.6(12)	4227(21)
$$^{129}$$Sn	2.23(4) min	$$3/2^+$$	0	$${}-80591(17) $$	–	–	4000.4(29)$$^a$$	4039(27)
$$^{129m}$$Sn	6.9(1) min	$$11/2^-$$	35.15(5)	$${}-80556(17) $$	–	–	4036.2(29)$$^a$$	4074(27)

## Results and discussion

**Table 2:** Potential candidate transitions from the initial states of the parent nucleus $^{129}$Sb to the excited final states of the daughter nucleus $^{129}$Te with ultra-low *Q* values. The first and second columns list the initial and final nuclear states, respectively. The third column gives the excitation energy $E_{x,d}$ of the final nuclear state and its uncertainty [49]. The fourth column specifies the decay type. The fifth and sixth columns present the derived $Q^{*}_{\beta }$ values (initial state decays to the excited final state of $^{129}$Te), taken from the literature (Lit.) [33, 49] and from this work (marked as JYFL), respectively. The seventh column shows the confidence level ($\sigma$) for the sign of $Q^{*}_{\beta }$. Spin-parity assignments and excitation energies enclosed in braces indicate uncertain values, which lead to corresponding uncertainties in the decay type. FU and FNU denote forbidden unique and forbidden non-unique $\beta$ decays, respectively

Initial state	Final state	$E_{x,d}$ (keV)	Decay type	$Q^{*}_{\beta }$ (Lit.)	$Q^{*}_{\beta }$ (JYFL)	$Q^*/\delta Q^*$ (JYFL)
$^{129}$Sb(7/2$^+$)	$^{129}$Te(3/2$^-$)	2360.472(21)	1st FU	15(21)	50.8(12)	42
$^{129}$Sb(7/2$^+$)	$^{129}\hbox {Te}(\{1/2^-\})$	2362.6(16)	{2nd FU}	13(21)	48.7(20)	24
$^{129}$Sb(7/2$^+$)	$^{129}$Te({3/2$^+$})	2370.5(16)	{2nd FNU}	5(21)	40.8(20)	20
$^{129}$Sb(7/2$^+$)	$^{129}$Te({1/2$^-$})	2377.4(16)	{2nd FU}	− 2(21)	33.9(20)	17
$^{129}$Sb(7/2$^+$)	$^{129}$Te(3/2$^-$)	2379.555(23)	1st FU	− 4(21)	31.7(12)	26
$^{129}$Sb$^*$(19/2$^-$)	$^{129}$Te({unknown})	4259.3(6)	–	− 33(21)	3.3(13)	2.5

**Fig. 4:**
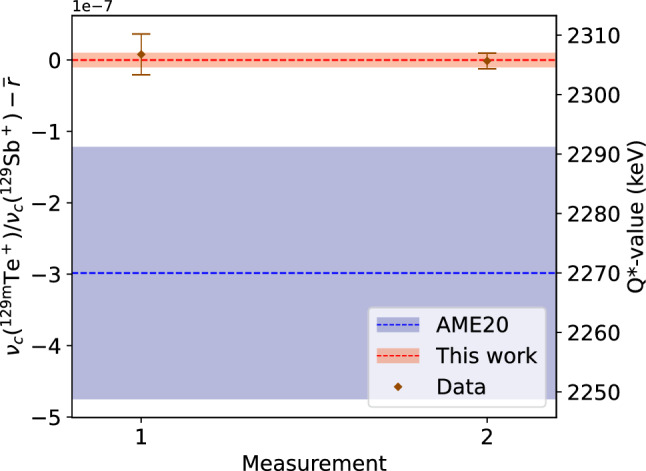
The deviation (left axis) of the individually measured cyclotron frequency ratios *r* ($\nu _c$($^{129m}$Te$^{+}$)/$\nu _c$($^{129}$Sb$^{+}$)) from the measured value $\overline{r}$ and (right axis) $Q^*$ values in this work compared to value adopted from AME2020 [33, 50]. The red points with uncertainties are measured individual data collected in two different time slots. The weighted average value from this work $\overline{r}$ = 1.000 019 203 (10) is illustrated by the horizontal dashed red line with its 1$\sigma$ uncertainty band. The dashed blue line is the value in AME2020 with its 1$\sigma$ uncertainty area shaded in blue

The measured frequency ratios $r = \nu _c$($^{129m}$Te$^{+}$)/$\nu _c$($^{129}$Sb$^{+}$) are presented in Fig. 4. The mass of $^{129}$Sb was determined from the frequency ratio using the standard relation for singly charged ions:

3 $$\begin{aligned} m_\text {ioi}=r(m_\text {ref}-m_e)+m_e \end{aligned}$$

where $m_\text {ioi}$ and $m_\text {ref}$ represent the masses of the ion of interest and the reference atoms, respectively, and $m_e$ is the mass of an electron. The electron binding energies of the ion of interest and the reference atoms are neglected in the calculation due to their small values (on the order of a few eV [51]), and *r* is close to 1.

For beta decays, which do not proceed between the ground states, we define the decay *Q* value as:

4 $$\begin{aligned} Q^*_\beta =Q_\beta +E_{x,p}-E_{x,d} \, \end{aligned}$$

where $Q_\beta$ is the ground-state-to-ground-state beta-decay *Q* value and $E_{x,p}$ and $E_{x,d}$ are the excitation energies of the initial and final states, respectively.

We directly determined the $Q^*_\beta$ between the $^{129}$Sb ground state and the isomeric state $^{129m}$Te used as a reference via:

5 $$\begin{aligned} Q^*_\beta = (m-m_\text {ref})c^2=[(r-1)(m_\text {ref}-m_e)]c^2. \end{aligned}$$

The measured $Q^*$ from the ground state to the 105.51 keV isomeric state is 2305.8(12) keV. By taking into account the excitation energy of the $^{129m}$Te, $E_{x,d}=105.51(3)$ keV, this yields $Q_{\beta }(^{129}$Sb$)=\varDelta E +E_x=2411.3(12)$ keV. The results are summarized in Table 1. The masses are given as mass-excess $ME=(m-Au)c^2$, where *A* is the mass number and *u* the atomic mass unit. The mass excess of $^{129m}$Sb was obtained using the literature value for its excitation energy and the determined ground-state mass excess. Both the mass excess and $Q_{\beta }$ values of $^{129}$Sb are 36(21) keV (1.7$\sigma$) greater and $\approx$ 18 times more precise than in AME20.

With the new precise ground-state-to-ground-state $Q_{\beta }=2411.3(12)$ keV, the ground-state-to-excited-state $Q^*_\beta$ ($=Q_{\beta }-E_x$) values for transitions to levels in $^{129}$Te are recalculated as listed in Table 2. All previously speculated ultra-low $Q^*_\beta$ branches have values greater than 31 keV, far outside the $<10$ keV regime required for competitive sensitivity in future electron antineutrino mass experiments. This definitively excludes $^{129}$Sb as a viable ultra-low-$Q^*$ candidate. For the $\beta ^-$-decay of isomer $^{129m}$Sb, with $Q_{\beta } = 4262.6(12)$ keV, the closest final state in $^{129}$Te is located at 4259.3(6) keV [11], yielding $Q^*=3.3(13)$ keV. However, the spin-parity of this level is unknown, and given the very high spin $J^\pi = 19/2^-$ for $^{129m}$Sb, only high-spin states are expected to be fed via $\beta$ decay. The level at 4259.3(6) keV in $^{129}$Te was observed in $^{128}$Te(*d*, *p*) transfer-reaction studies [52] that are unlikely to populate such high-spin states. Thus, $^{129m}$Sb is also not an ultra-low $\beta ^-$-decay *Q* value emitter.

The revised $Q_\beta$ values for $^{129}$Sb, $^{129m}$Sb, $^{129}$Sn, and $^{129m}$Sn (Table 1), obtained by combining recent CPT measurements of $^{129}$Sn [48] with the results from this work, substantially reduce the uncertainties in the $A=129$ decay chain. These improvements affect *r*-process nucleosynthesis models, especially the amount and timing of energy released by long-lived isomeric states (astromers) that influence kilonova light curves [8, 9]. Compared to AME2020, the slightly lower *Q* values mean a modest decrease in total energy release, which could lead to small changes in the predicted brightness and evolution of kilonova electromagnetic signals. Additionally, the demonstrated clean production and separation of both ground and isomeric states of $^{129}$Sb via proton-induced fission, combined with the precise isomer mass, strengthens the feasibility of future NEEC studies in highly charged ions at IGISOL [3, 13, 14, 19, 20].

## Conclusions

We have performed the first direct high-precision measurement of the $Q_\beta$ value of $^{129}$Sb and its long-lived isomer using JYFLTRAP, achieving a $Q_\beta$ precision of 1.2 keV for the ground state – a factor of $\sim$ 18 improvement over AME2020. The resulting $Q_\beta = 2411.3(12)$ keV shifts previously proposed ultra-low-$Q^*$ $\beta ^-$ branches to 31–51 keV, definitively ruling them out as candidates for future electron antineutrino mass experiments. The isomer *Q* value of 4262.6(12) keV and improved mass excess also enhance constraints on the $A=129$ decay chain, with implications for *r*-process nucleosynthesis and kilonova electromagnetic signatures. In addition, coupling the first trap buffer-gas cleaning with the PI-ICR method, significantly enhances the capabilities of JYFLTRAP for rapid preparation of mono-isomeric and mono-isotopic ions, facilitating high-precision mass measurements and post-trap decay spectroscopy applications such as NEEC.

## Data Availability

This manuscript has associated data in a data repository. [Author’s comment: The datasets generated during and/or analysed during the current study are available in the IDA Fairdata repository, https://doi.org/10.23729/fd-2d319d81-c747-33a9-9627-4bf3a75202bd.]

## Code Availability Statement

This manuscript has no associated code/software. [Author’s comment: Code/Software sharing not applicable to this article as no code/software was generated or analysed during the current study.]
